# *GSTU43* gene involved in ALA-regulated redox homeostasis, to maintain coordinated chlorophyll synthesis of tomato at low temperature

**DOI:** 10.1186/s12870-019-1929-1

**Published:** 2019-07-18

**Authors:** Tao Liu, Qingjie Du, Suzhi Li, Jianyu Yang, Xiaojing Li, Jiaojiao Xu, Pengxiang Chen, Jianming Li, Xiaohui Hu

**Affiliations:** 1 College of Horticulture, Northwest A & F University, Yangling, 712100 Shaanxi China; 2 Key Laboratory of Protected Horticultural Engineering in Northwest, Ministry of Agriculture, Yangling, 712100 Shaanxi China; 3 Shaanxi Protected Agriculture Research Centre, Yangling, 712100 Shaanxi China

**Keywords:** 5-aminolevulinic acid (ALA), Antioxidant, Chlorophyll synthesis, Glutathione S-transferase (*GSTU43*) gene, Low temperature, *Solanum lycopersicum* (tomato)

## Abstract

**Background:**

Exogenous 5-aminolevulinic acid (ALA) positively regulates plants chlorophyll synthesis and protects them against environmental stresses, although the protection mechanism is not fully clear. Here, we explored the effects of ALA on chlorophyll synthesis in tomato plants, which are sensitive to low temperature. We also examined the roles of the glutathione S-transferase (*GSTU43*) gene, which is involved in ALA-induced tolerance to oxidation stress and regulation of chlorophyll synthesis under low temperature.

**Results:**

Exogenous ALA alleviated low temperature caused chlorophyll synthesis obstacle of uroporphyrinogen III (UROIII) conversion to protoporphyrin IX (Proto IX), and enhanced the production of chlorophyll and its precursors, including endogenous ALA, Proto IX, Mg-protoporphyrin IX (Mg-proto IX), and protochlorophyll (Pchl), under low temperature in tomato leaves. However, ALA did not regulate chlorophyll synthesis at the level of transcription. Notably, ALA up-regulated the *GSTU43* gene and protein expression and increased GST activity. Silencing of *GSTU43* with virus-induced gene silencing reduced the activities of GST, superoxide dismutase, catalase, ascorbate peroxidase, and glutathione reductase, and increased the membrane lipid peroxidation; while fed with ALA significant increased all these antioxidase activities and antioxidant contents, and alleviated the membrane damage.

**Conclusions:**

ALA triggered GST activity encoded by *GSTU43*, and increased tomato tolerance to low temperature-induced oxidative stress, perhaps with the assistance of ascorbate- and/or a glutathione-regenerating cycles, and actively regulated the plant redox homeostasis. This latter effect reduced the degree of membrane lipid peroxidation, which was essential for the coordinated synthesis of chlorophyll.

**Electronic supplementary material:**

The online version of this article (10.1186/s12870-019-1929-1) contains supplementary material, which is available to authorized users.

## Background

Plants exposed to low temperature exhibit a short-term stress response involved modification of gene expression and levels of plant hormones, increased cross-talk signaling, and accumulation of osmolytes and antioxidants, presumably to protect against long-term damage [1]. Extended exposure to low temperature damages DNA and protein, causes lipid peroxidation, and results in general physiological and metabolic disturbances that reduce growth and vigor [2].

It is well-known that exogenous application of 5-aminolevulinic acid (ALA) improves plant tolerance to environmental stress [3–9]. This compound is also the precursor to porphyrins, which are, in turn, the precursors to plant pigments, including heme and chlorophyll; as such, application of ALA increased the chlorophyll content of plants [8, 10, 11]. In a previous study, we observed that ALA improved perception of, and tolerance to, oxidative stress imposed by low temperature. We attributed this to the accumulation of glutathione (GSH) and ascorbate (AsA) [12].

The tripeptide glutathione (GSH; composed of γ-Glu-Cys-Gly) is an antioxidant with a central role in the regulation of redox balance and signaling [13–16]. Plants contain several GSH-dependent detoxifying enzymes [17, 18], most notably glutathione S-transferases (EC 2.5.1.18, GSTs). GSTs are an evolutionarily ancient, large, and diverse family. GSTs have catalytic functions and non-catalytic functions (such as binding and transport of secondary metabolites and hormones), which related to their structural dynamics [19].

Many studies have shown that GSTs play crucial roles in mediating plant perception of, and tolerance to, abiotic stress. For example, induction of *GST*s by salicylic acid (SA) [20] or their over-expression [21] sustained tomato redox homeostasis when plants were exposed to salt stress. Also, when the drought-tolerant *Prosopis juliflora* (*PjGSTU1*) gene was introduced into tobacco, its peroxidase activity reduced excess reactive oxygen species (ROS) and maintained the redox balance in drought-stressed plants [22]. In other studies, the over-expression of a tobacco U class of GSTs elevated the GST activity, apparently increasing the levels of antioxidants sufficiently to permit tobacco seedlings [23] to cope with salt and chilling stress and to permit rice plants to cope with low temperature stress [24]. The non-catalytic functions of GSTs may also be important in stress tolerance. GSTs could bind and/ or conjugate metabolites such as porphyrins [25], phytohormones [26], and other secondary metabolites [27], and then transport them to perform function or protect against cellular oxidation [19].

The metabolism of porphyrins, central to the synthesis of chlorophyll and heme, is carefully regulated in the chloroplast. In healthy cells, the integrated membrane system and conjugated protein provide guarantee for chlorophyll synthesis [28]. However, the membranes and conjugated protein of stressed cells may be damaged by peroxides, and under this circumstance porphyrins could leak into the cytosol [19, 25, 29]. If so, they would be oxidized to the lipophilic and phytotoxic protoporphyrin, exacerbating the already damaging effect of peroxide accumulation in stressed cells [25, 29]. The ZmGSTU1 of maize, bound porphyrinogens that had leaked from chloroplasts under oxidative stress, preventing their auto oxidation and apparently protecting the cells from oxidative stress [29].

Tomatoes are sensitive to low temperatures (8 °C–15 °C), in the winter and early spring in China. Low temperature can severely limit the growth and yield of tomatoes. Many studies have illustrated that exogenously applied ALA reduced ROS accumulation by increasing antioxidant ability under abiotic stress [30–33]. Our preliminary studies showed that ALA induced early H_2_O_2_ signaling under normal conditions, which then interacted with JA, induced the downstream NO, regulated the redox state, resulting in elevated antioxidant capacity and photosynthesis in tomato plants under low temperature [12, 34]. This was accompanied by ALA-induced up-regulation of the *GSTU43* gene (Additional file 2: Fig. S1), which encodes a GST. These results inspired us to ask: What is the relationship between redox homeostasis and porphyrin synthesis regulated by exogenous ALA during low temperature stress? What is the role of *GSTU43* in the maintenance of redox homeostasis at low temperature? So, in this study, we first assessed the effect of exogenous ALA on chlorophyll synthesis and GST activity encoded by *GSTU43* in tomato leaves. And we further explored whether *GSTU43* involved in ALA-mediated redox status and alleviation of membrane lipid peroxidation, which was crucial for the coordinated chlorophyll synthesis.

## Methods

### Treatment with exogenous ALA and Gabaculine

Tomato (*Solanum lycopersicum* cv. Jinpeng no. 1, which is sensitive to low temperature stress) seeds (purchase from Xi’an Jinpeng Seedlings Co., Ltd. Shaanxi, China.) were used in this study. Seedlings cultivation was according to our previous study [12]. When the fifth true leaves were completely expanded, plants of similar appearance were selected for the experiments.

We wished to compare the effects of exogenous ALA on chlorophyll synthesis, expression of *GSTU43* and GST activity in plants exposed to low and normal temperature. Plants were sprayed with 6 mL either distilled water or 25 mg·L^− 1^ ALA (Sigma-Aldrich, St. Louis, MO, USA) solution [12], containing a few drops of Tween-20, 1.5 h before the night. Twelve hours later, half of ALA-treated and untreated plants were kept at normal temperatures (25 °C/18 °C, day/night) while the rest of the plants were exposed to a low-temperature regime: 15 °C during the photoperiod and 8 °C during the dark, with the same relative humidity and light period as the normal-temperature plants.

To determine whether exogenous ALA-induced *GSTU43* up-regulation and GST activity is due to endogenous ALA. The tomato leaves were pretreated with 100 μM Gabaculine (GAB, inhibition of ALA synthesis that inactivates glutamate-1-semialdehyde aminotransferase) [35, 36]. After 8 h, the leaves were sprayed with distilled water or 25 mg·L^− 1^ ALA. Twelve hours later, the plants were exposed to low temperatures. After 24 h low temperatures, the *GSTU43 gene* expression and protein levels, and GST activity were measured.

### VIGS plants and experiments

To prepare the pTRV2-*GSTU43* vectors, a 294 bp fragment of *GSTU43* was amplified with PCR using the forward primer GCTCTAGAATGCCAGTAATGGGGAAAGC and the reverse primer GGGGTACCTCTTGGCGGTAAATATTCCTTG; the primers contained XbaI and KpnI restriction sites. The PCR fragment was inserted into the XbaI and KpnI site of pTRV2 vector. The pTRV2-*GSTU43* VIGS constructs were confirmed by sequencing. Then, *Agrobacterium tumefaciens* strain GV3101 was transformed with pTRV2-*GSTU43* as described by Cheng et al. [37]. *Agrobacterium*-mediated virus infection was performed as described of Ekengren et al. [38], when two cotyledons of tomato seedlings (cv. Jinpeng no. 1), cultivated as our previous study [12], completely expanded. An *Agrobacterium* culture carrying an empty pTRV2 vector was also infiltrated into a set of plants that served as a control. The inoculated plants were maintained at 20–22 °C in a growth chamber with relative humidity of 60% ± 5%, and a two-phase photoperiod of 12.5 h with a PPFD of 350 μmol photons·m^− 2^·s^− 1^ followed by 1.5 h with a PPFD of 50 μmol photons·m^− 2^·s^− 1^); the photoperiod was followed by a 10-h dark period. The temperature and light period were set according to the description of Cheng et al. [37] and our preliminary experiment. After 35 days, the gene silencing efficiency was assessed with quantitative real-time PCR (qRT-PCR) before the plants were used in assays. And then, plants of similar appearance were selected for the experiments.

The plants were pre-treated with exogenous ALA, then exposed to normal condition (22 °C/20 °C, day/night) or low temperature (15 °C/8 °C, day/night) 12 h later. After 24 h of low temperature, malondialdehyde (MDA) content, relative electrical conductivity (REC), H_2_O_2_ content, maximal quantum yield of PSII photochemistry (Fv/Fm), antioxidase and antioxidants levels, expression of *GSTU43* gene of VIGS plants were measured. Frozen samples were collected for the measurement of MDA, H_2_O_2_, antioxidases, antioxidants, and gene expression, while fresh samples were used for the REC and Fv/Fm analysis. Samples for plant dry weight were taken 6 d after the start of the low temperature treatment.

Leaf samples were collected at different times after the start of the temperature treatment, frozen immediately in liquid nitrogen, and stored at − 80 °C until needed. Fresh leaf samples were harvested for measuring the chlorophyll and related compounds, plant dry weight, and Fv/Fm and REC. All the experiments were repeated for three times, and three independent biological replicates for each time.

### Measurement of plant dry weight

Plants were rinsed three times with distilled water, oven dried at 105 °C for 15 min, and then at 75 °C for 72 h to obtain dry weights.

### Chlorophyll synthesis analysis

Chlorophyll was extracted from the fifth fully expanded leaf in acetone, ethanol, and water (4.5: 4.5: 1, v/v/v) and pigments were analyzed spectrophotometrically based on the absorbance at 450, 645, and 663 nm [39]. The content of ALA was determined according to the methods of Morton [40]. The porphobilinogen (PBG) and UROIII content were measured according to the methods of Bogorad [41]. The Proto IX, Mg-protoporphyrin IX (Mg-proto IX) and protochlorophyll (Pchl) were all carried out according to the methods of Hodgins and Van Huystee [42]. All the details for determining these chlorophyll precursors above were described in our previous studies [43].

### Membrane lipid peroxidation assays

The H_2_O_2_ content was obtained via monitoring the absorbance at 412 nm [44], as described in our previous study [12]. Using the methods of Hodges et al. [45], and Zhou and Leul [46] to determine the MDA content and REC, respectively. Fv/Fm was assayed as described in our previous study [12, 47], and the plants were subjected to dark for 30 min before determination.

### Antioxidant assays

The activity of superoxide dismutase (SOD; EC 1.15.1.1) was conducted using the method of Giannopolitis and Ries [48]. The activities of catalase (CAT; EC 1.11.1.6), glutathione reductase (GR; EC 1.6.4.2), ascorbate peroxidase (APX; EC 1.11.1.11), monodehydroascorbate reductase (MDHAR; EC 1.6.5.4), dehydroascorbate reductase (DHAR; EC 1.8.5.1), and the contents of glutathione and ascorbate were assayed as described in our previous study [12, 49]. The GST activity was measured using GSH-ST assay kit (Nanjing Jiancheng Bioengineering Institute, Nanjing, China) following the supplier’s instruction.

### Gene expression analyses

For RNA sequencing experiments, the tomato leaves, treated with ALA or/ and temperature, were collected and frozen immediately in liquid nitrogen, and stored at − 80 °C until needed. And then, the RNA sequencing were carried out using Illumina High-Seq platform at Biomarker Technologies Corporation (Beijing, China). The total RNA extracted and qRT-PCR analyses were conducted according to our previous descriptions [34] using two different reference genes, *actin7* and *GAPDH* [50], and the relative gene expression was calculated following Livak and Schmittgen [51]. The gene specific primers are listed in Additional file 1: Table S1.

### Protein extraction and western blotting

The protein extraction and western blotting according to the methods of Zhou et al. [52] with a modification. Briefly, for protein extraction, tomato leaves, collected 24 h after low temperature treatment, were ground in liquid nitrogen, and homogenized in extraction buffer (50 mM Tris-HCl, pH 7.5, containing, 150 mM NaCl, 2 mM EDTA-Na_2_, 10% glycerol, 1% Triton X-100, 1 mM PMSF and 1 mM DTT). The extracts were centrifuged at 12,000 g for 20 min, and the extracted protein was heated at 95 °C for 15 min, then separated using 10% SDS-PAGE. For western blotting, the proteins on the SDS-PAGE gel were transferred to a polyvinylidene-fluoride membrane. The membrane was blocked for 12 h at 4 °C in 1 × BLOK BSA in PBS [Sangon Biotech (Shanghai) Co., Ltd], then incubated for 4 h in antibody dilution buffer and a rabbit anti-GSTU43 polyclonal antibody [Genscript (Nanjing) Co., Ltd.], or a rabbit antiactin polyclonal antibody (Abcam). And then the membrane was incubated with a goat anti-rabbit HRP-conjugated antibody (Cell Signaling Technology). Accumulation of actin and GSTU43 were quantified using Image Lab 6.0 software (Bio-Rad, Hercules, CA, USA).

### Statistical analysis

All data were subjected to analysis of variance (ANOVA) using SAS 8.0 software (SAS Institute, Cary, NC, USA). Significant difference was assessed using Tukey’s test at a significance level of *P <* 0.05, unless stated otherwise.

## Results

### ALA regulated chlorophyll synthesis in low temperature-stressed tomato leaves

**Fig. 1:**
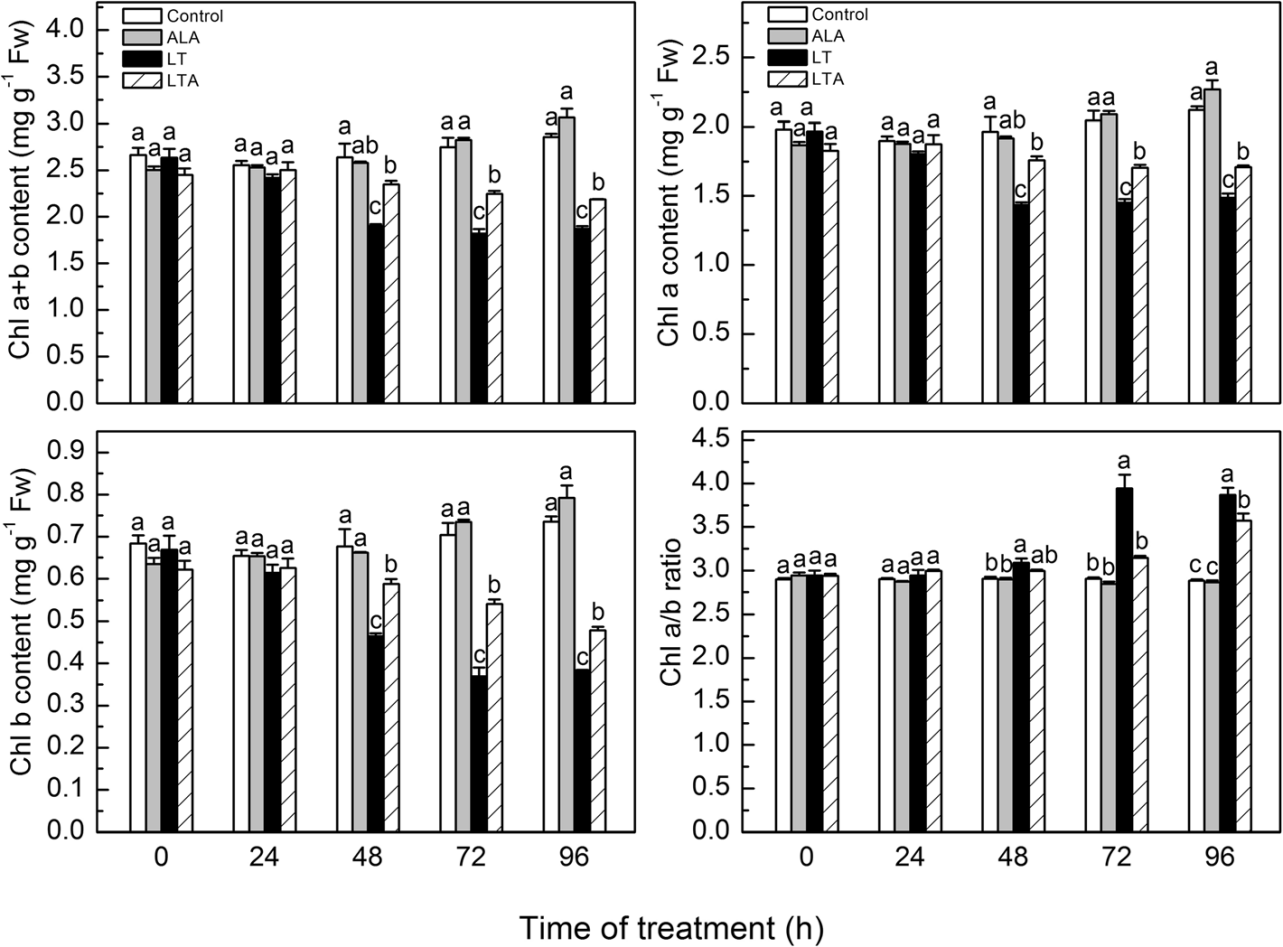
ALA induced kinetic changes of chlorophyll content in tomato leaves within 96 h after imposition of low temperature. The tomato leaves treated with distilled water or 25 mg·L^− 1^ ALA then exposed to normal condition (control and ALA) or low temperature (LT and LTA) 12 h later. The chlorophyll (Chl) content were measured when the low temperature started. Data are expressed as the mean ± standard error of three independent biological replicates. The experiments were repeated for three times. Different letters above the bars indicate a significant difference determined by one-way ANOVA with Tukey’s test (*P <* 0.05). Fw, fresh weight

**Fig. 2:**
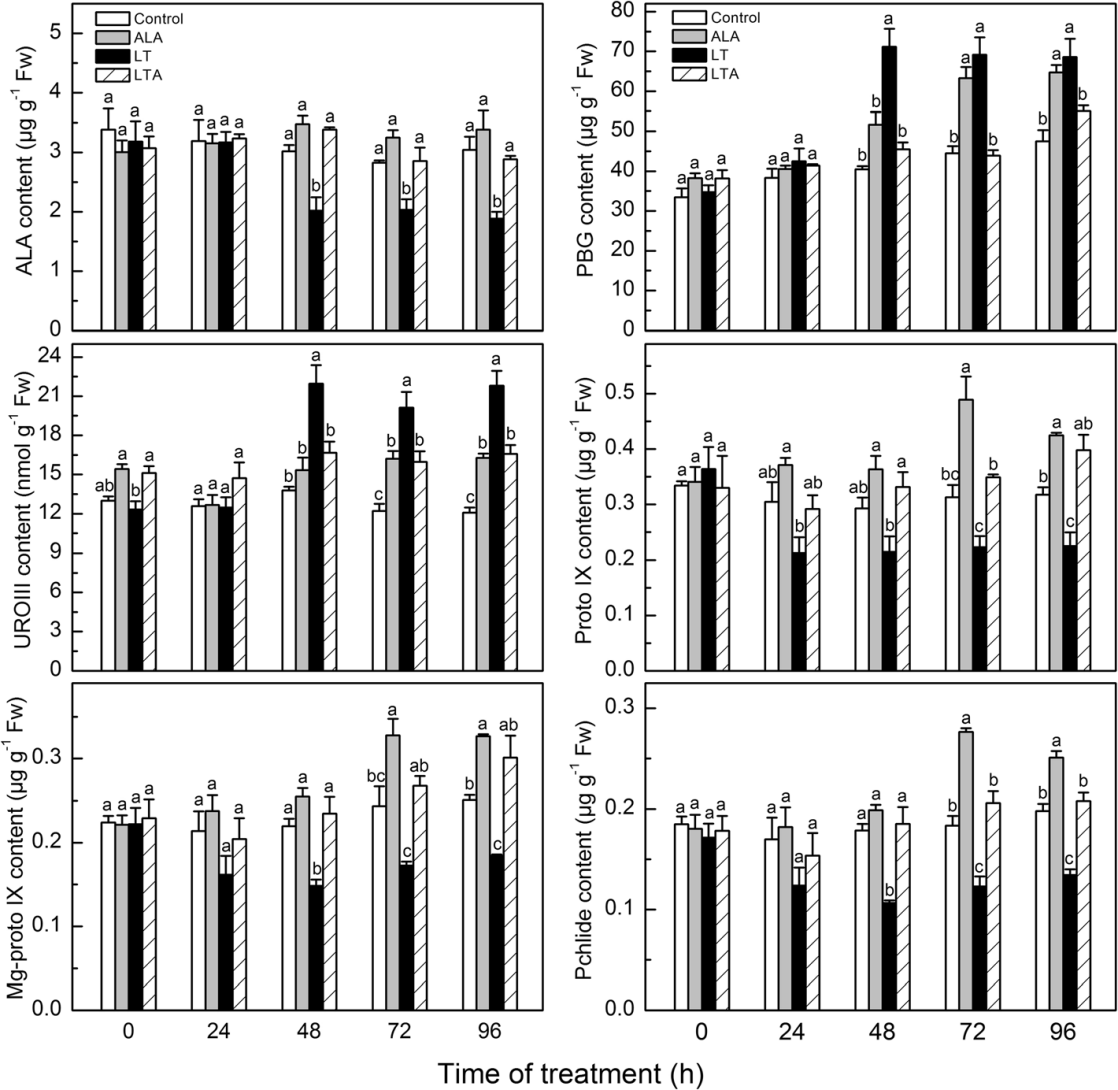
ALA induced kinetic changes of chlorophyll precursors in the chlorophyll biosynthetic pathway in tomato leaves within 96 h after imposition of low temperature. The tomato leaves treated with distilled water or 25 mg·L^− 1^ ALA then exposed to normal condition (control and ALA) or low temperature (LT and LTA) 12 h later. The precursor content were measured when the low temperature started. Data are expressed as the mean ± standard error of three independent biological replicates. The experiments were repeated for three times. Different letters above the bars indicate a significant difference determined by one-way ANOVA with Tukey’s test (*P <* 0.05). ALA, 5-aminolevulinic acid; PBG, porphobilinogen; UROIII, uroporphyrinogen III; Proto IX, protoporphyrin IX; Mg-proto IX, Mg-protoporphyrin IX; and Pchl, protochlorophyll

ALA is a precursor in the chlorophyll biosynthetic pathway. Exogenous application of ALA has been shown to increase the chlorophyll content in leaves of crop species [6, 33, 53], so we began this study by measuring the effect of exogenous ALA on the chlorophyll content and other related compounds (precursors) in low temperature-stressed tomato leaves compared to leaves from unstressed plants (Figs. 1 and 2).

In plants grown in normal temperature (25 °C/18 °C), exogenous ALA had no obvious effect on either chlorophyll or endogenous ALA levels, while there were more PBG, UROIII, Proto IX, Mg-proto IX, and Pchl at 72 and 96 h in ALA-treated plants than in untreated controls. Low temperature treatment (15 °C/8 °C) by itself decreased the amounts of chlorophyll and most of the precursors, but dramatically increased PBG, UROIII, and the ratio of Chl a/b, apparent 48 h after treatment was started. However, when low temperature-stressed plants received exogenous ALA, there was markedly less PBG and UROIII, and more of the other precursors and chlorophyll after 48 h than the low temperature-stressed plants alone.

**Fig. 3:**
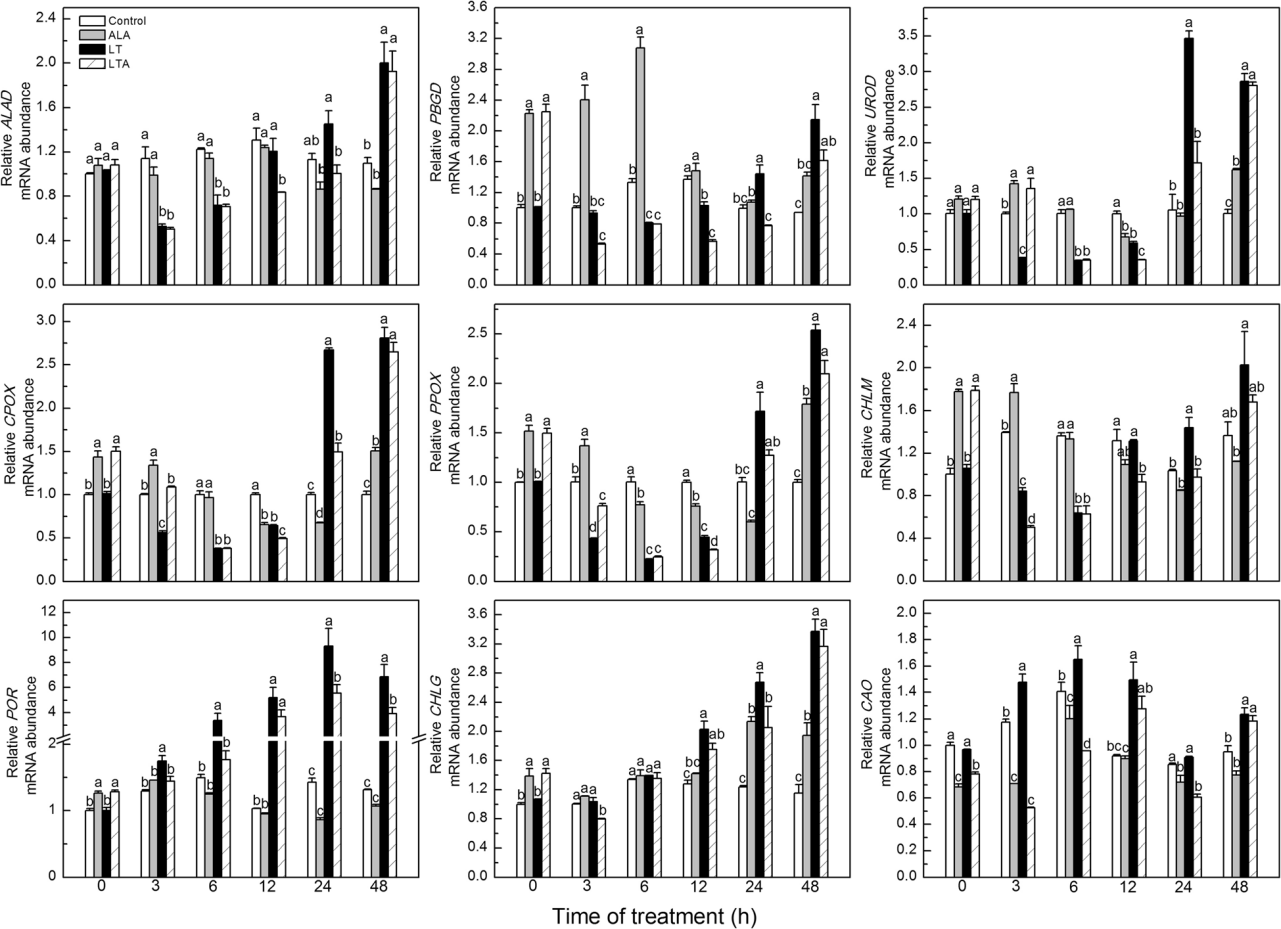
ALA induced kinetic changes of gene expression in chlorophyll synthesis with tomato leaves under low temperature within 48 h. The tomato leaves treated with distilled water or 25 mg·L^− 1^ ALA then exposed to normal condition (control and ALA) or low temperature (LT and LTA) 12 h later. The expression of genes were carried out when the low temperature started. The gene transcription levels in control plants at 0 h was normalized as 1. Data are expressed as the mean ± standard error of three independent biological replicates. The experiments were repeated for three times. Different letters above the bars indicate a significant difference determined by one-way ANOVA with Tukey’s test (*P <* 0.05). *ALAD*, aminolevulinic dehydratase gene; *PBGD*, porphobilinogen deaminase gene; *UROD*, uroporphyrinogen decarboxylase gene; *CPOX*, coproporphyrinogen III oxidase gene; *PPOX*, protoporphyrinogen IX oxidase gene; *CHLM*, Mg-protoporphyrin IX methyltransferase gene; *POR*, protochlorophyllide oxidoreductase gene; *CHLG*, chlorophyll synthase gene; *CAO*, chlorophyllide a oxygenase gene

We performed qRT-PCR to analyze the genes expression of the key enzymes involved in chlorophyll biosynthetic pathway. Under normal condition, application of ALA had no obvious effect on aminolevulinic dehydratase (*ALAD*) expression at any time, but dramatically elevated the transcription of other genes at different times: specifically, expression of porphobilinogen deaminase (*PBGD*) was elevated before 6 h, uroporphyrinogen decarboxylase (*UROD*) at 3 h and 48 h, coproporphyrinogen III oxidase (*CPOX*) and protoporphyrinogen IX oxidase (*PPOX*) at 0 h, 3 h, and 48 h, Mg-protoporphyrin IX methyltransferase (*CHLM*) within 3 h, protochlorophyllide oxidoreductase (*POR*) at 0 h, and chlorophyll synthase (*CHLG*) at 0 h, 24 h, and 48 h (Fig. 3). Expression of chlorophyllide a oxygenase (*CAO*) at 0 h, 3 h, 6 h, and 24 h, *UROD* at 12 h, *CPOX* at 12 h and 24 h, PPOX at 6 h and 12 h, respectively, in ALA-treated leaves were lower than in the control. When exogenous ALA was supplied before low temperature stress, the genes expression of *PBGD*, *CPOX*, *PPOX*, *CHLM*, *POR,* and *CHLG* were significantly up-regulated at 0 h, and then decreased expression of these genes after 3 h, while *UROD* transcript was up-regulated at 3 h and down-regulated at 12 h and 24 h, respectively, compared to low temperature-stressed plants alone.

These results showed that low temperature disrupted chlorophyll synthesis and hindered URO III conversion to Proto IX, and that exogenous ALA counteracted the disruptive effect of low temperature. However, this pattern was not apparent in gene expression, indicating that ALA may regulate chlorophyll synthesis at a level other than transcription [54, 55].

### ALA positively induced the GSTU43 gene and protein expression, and GST activity in tomato at low temperature

**Fig. 4:**
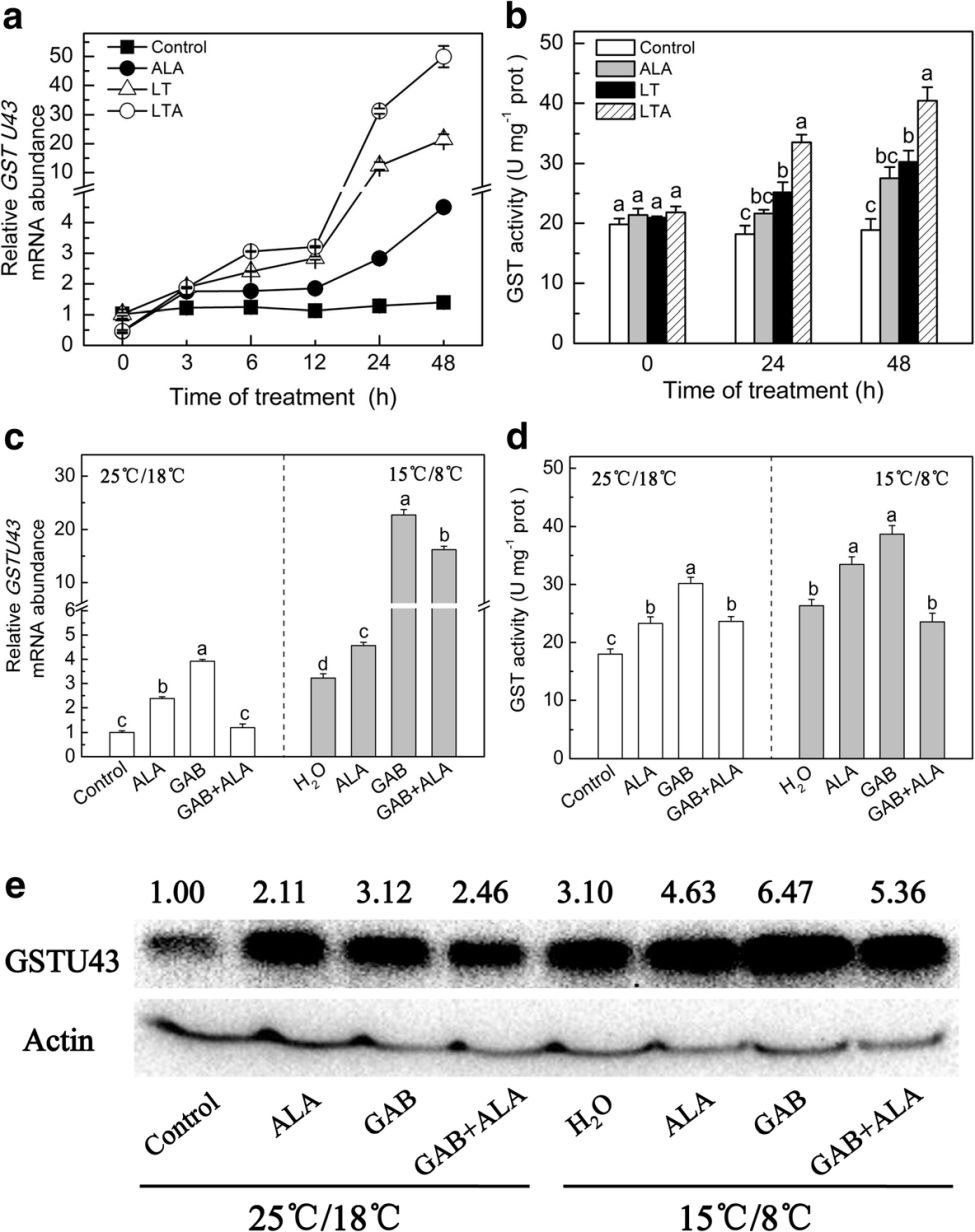
ALA induced changes of glutathione S-transferase (*GSTU43*) gene and protein expression and GST activity in tomato leaves under low temperature within 48 h. The tomato leaves treated with distilled water or 25 mg·L^− 1^ ALA then exposed to normal condition (control and ALA) or low temperature (LT and LTA) 12 h later. The expression of *GSTU43* (the gene transcription levels in control plants at 0 h was normalized as 1) (**a**) and GST activity (**b**) were carried out when the low temperature started. The tomato leaves were pretreated with 100 μM Gabaculine (GAB, inhibition of ALA synthesis that inactivates glutamate-1-semialdehyde aminotransferase). After 8 h, the leaves were sprayed with distilled water or 25 mg·L^− 1^ ALA. Twelve hours later, the plants were exposed to low temperatures. After 24 h low temperatures, the *GSTU43 gene* expression (the gene transcription levels in control plants at 24 h was normalized as 1) (**c**) and GST activity (**d**), and protein levels (**e**) were measured. Data are expressed as the mean ± standard error of three independent biological replicates. The experiments were repeated for three times. Different letters above the bars indicate a significant difference determined by one-way ANOVA with Tukey’s test (*P <* 0.05)

Glutathione S-transferases regulate the redox balance and improve plant oxidation resistance [19]. We observed that *GSTU43* was significantly up-regulated by ALA using RNA-seq in tomato (Additional file 2: Figure S1), and we also verified the expression of *GSTU43* and its associated GST activity. In this study, ALA significantly raised the *GSTU43* expression since 6 h, and increased GST activity was apparent at 24 h and 48 h (Fig. 4a and b). This elevation was apparent in both temperature treatments. In normal temperatures, ALA increased *GSTU43* transcription by 121 and 224%, and the activity of GST by 18.9 and 45.9%, at 24 h and 48 h, respectively. When the plants were low temperature-stressed, *GSTU43* expression was increased 154 and 132%, and GST activity was increased 33.2 and 33.6% at 24 h and 48 h, respectively.

Compared with control plants under normal or low temperature stress alone, fed with ALA dramatically improved *GSTU43* gene and protein expression, and GST activity (Fig. 4c, d and e). Compared with control plants, GAB treatment further provoked a significant increase of *GSTU43* gene and protein expression, and GST activity by 290, 212 and 67.8%, respectively, under normal temperature; while GAB plus low temperature triggered an increase of *GSTU43* gene and protein expression, and GST activity 603, 108 and 46.9% increase, respectively, compared to low temperature treatment alone. GAB plus ALA treatment dramatically decreased the GAB triggered *GSTU43* gene and protein expression, and GST activity, under normal or low temperature.

### GSTU43 is involved in ALA- induced low temperature tolerance

We silenced *GSTU43* with VIGS to assess the function of *GSTU43* in ALA-induced low temperature tolerance. The average silence efficiency was about 68.9% (Additional file 3: Figure S2a). Under low temperature, the dry weight of low temperature-stressed, *GSTU43* silenced plants was 15.0% less than the pTRV plant, and supplementing the *GSTU43* silenced, low temperature-stressed plants with ALA restored the dry weight to the pTRV level (Additional file 3: Figure S2b).

**Fig. 5:**
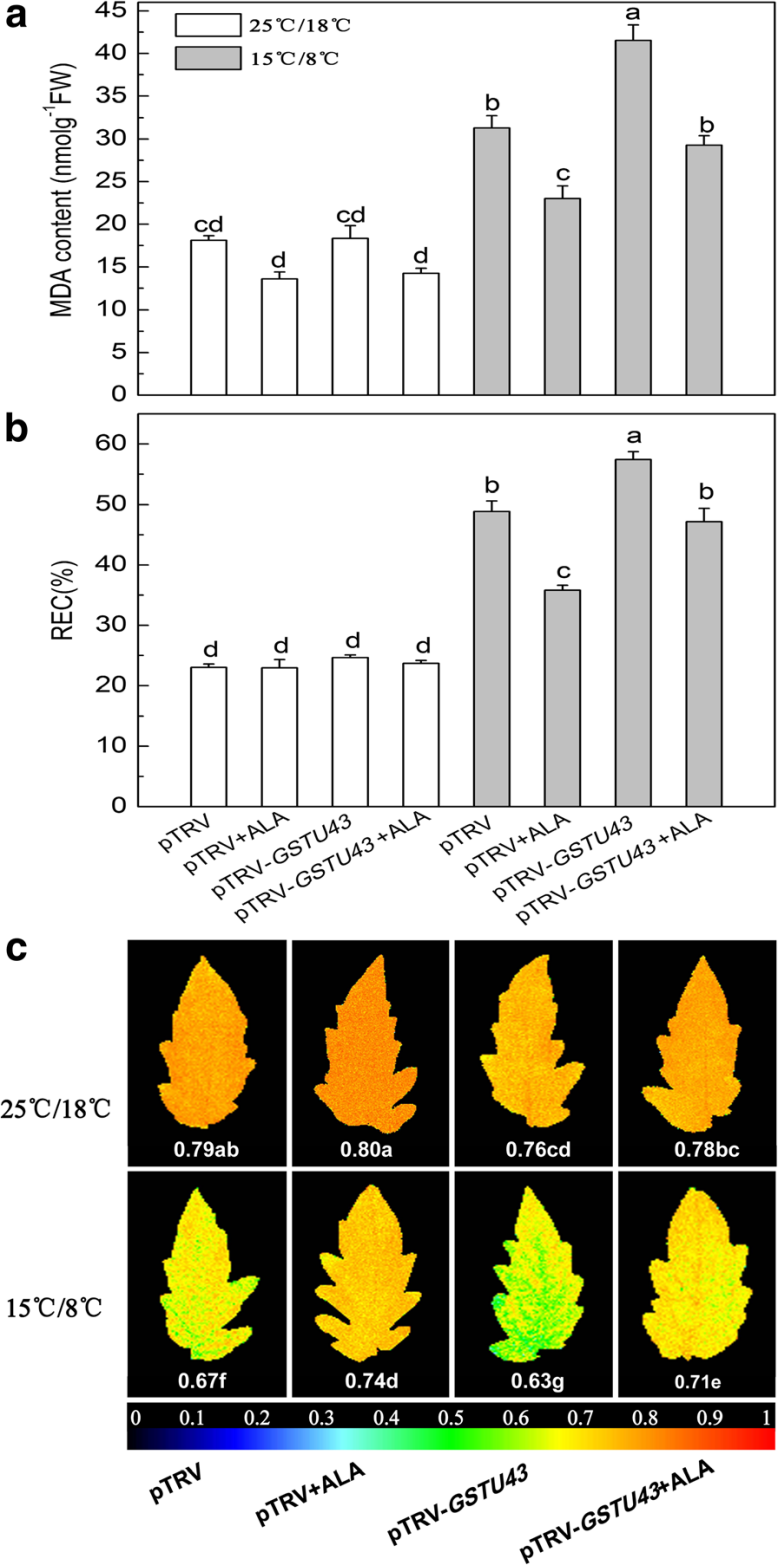
Effects of exogenous ALA on membrane peroxidation injury in partial *GSTU43*-silenced tomato leaves under low temperature. **a** malondialdehyde (MDA) levels, (**b**) relative electrical conductivity (REC), (**c**) maximal quantum yield of PSII photochemistry (Fv/Fm). The VIGS plants leaves were treated with distilled water or 25 mg·L^− 1^ ALA, then exposed to normal condition (22 °C/20 °C, day/night) or low temperature (15 °C/8 °C, day/night) 12 h later. After 24 h of low temperature, these indexes were measured. Data are expressed as the mean ± standard error of three independent biological replicates. The experiments were repeated for three times. Different letters above the bars indicate a significant difference determined by one-way ANOVA with Tukey’s test (*P <* 0.05)

We also assessed membrane lipid peroxidation damage. We observed no effect of exogenous ALA on levels of MDA and REC, or on Fv/Fm in pTRV-*GSTU43* plants grown at normal temperatures (Fig. 5). Low temperatures resulted in accumulation of MDA, elevated REC, and reduced Fv/Fm compared to normal temperature. The differences were most striking in the low temperature-stressed, pTRV-*GSTU43* plants; ALA reversed the effects of silencing and restored the values to those of the pTRV low temperature-stressed plants (Fig. 5).

### Silencing of GSTU43 compromised ALA- induced enhancement in antioxidant capacity

**Fig. 6:**
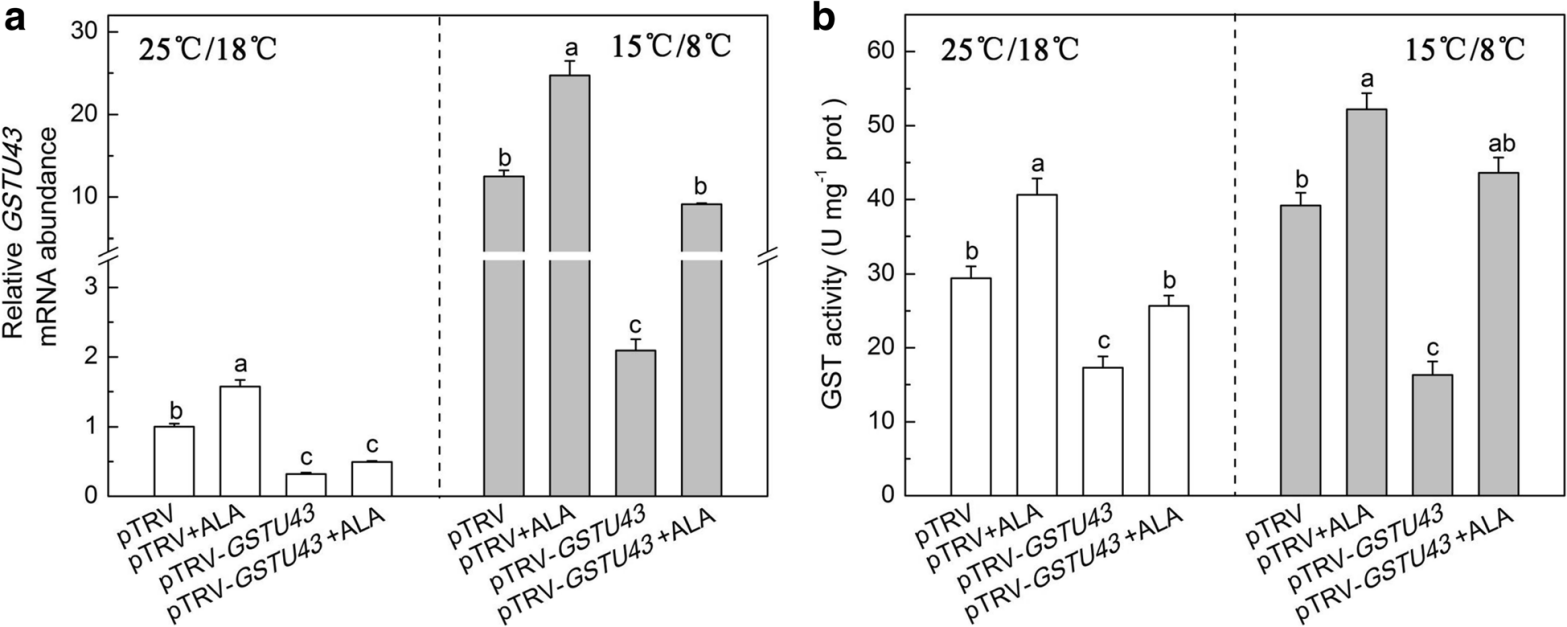
ALA induced *GSTU43* expression and GST activity in partial *GSTU43*-silenced plants exposed to low temperature. **a** *GSTU43* expression. The gene transcription levels in pTRV plants under normal condition at 24 h was normalized as 1, (**b**) GST activity. The VIGS plants leaves were treated with distilled water or 25 mg·L^− 1^ ALA, then exposed to normal condition (22 °C/20 °C, day/night) or low temperature (15 °C/8 °C, day/night) 12 h later. After 24 h of low temperature, these indexes were measured. Data are expressed as the mean ± standard error of three independent biological replicates. The experiments were repeated for three times. Different letters above the bars indicate a significant difference determined by one-way ANOVA with Tukey’s test (*P* < 0.05)

Redox homeostasis is important for plant growth and development [56, 57], and we have shown that ALA positively regulates the redox state of tomato via the accumulation of GSH and AsA, mediated by H_2_O_2_ [12]. We further investigated the role of *GSTU43* in ALA-induced oxidative stress tolerance of tomato under low temperature by assessing the expression of *GSTU43*, and the level of antioxidant and activities of antioxidases. Under normal temperatures, ALA treated plants showed higher *GSTU43* expression and GST activity than the plants without ALA treated (Fig. 6).

We next examined these parameters in low temperature-stressed plants. Expression of *GSTU43* and GST activity in pTRV-*GSTU43* plants exposed to low temperature was significantly less than in low temperature-stressed pTRV plants. In low temperature-stressed pTRV-*GSTU43* plants treated with ALA, *GSTU43* expression increased by 335%, and GST activity increased by 167%, respectively, compared to pTRV-*GSTU43* plants not receiving ALA.

**Fig. 7:**
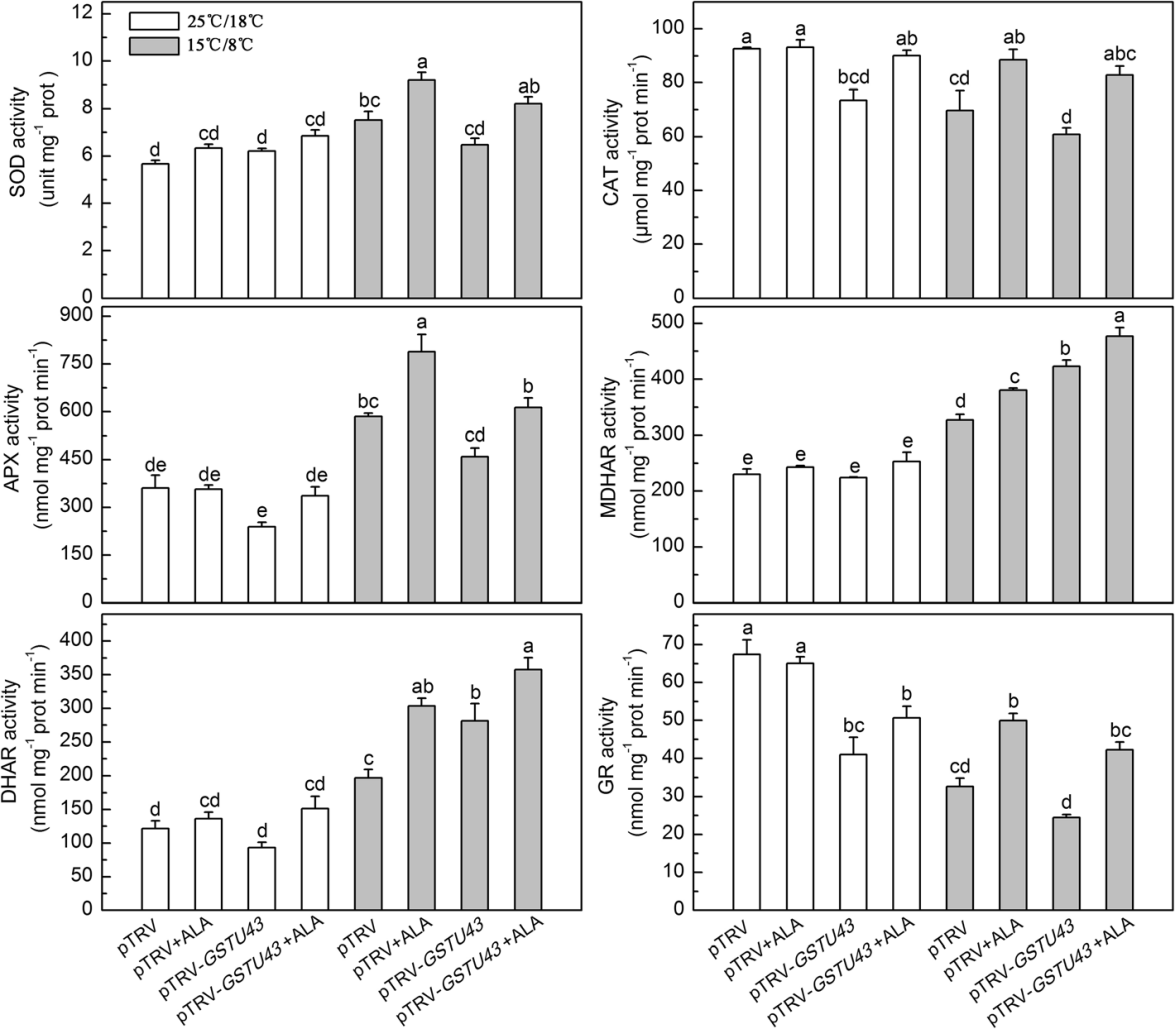
Effects of exogenous ALA on antioxidase activities in partial *GSTU43*-silenced plants under low temperature. The VIGS plants leaves were treated with distilled water or 25 mg·L^− 1^ ALA, then exposed to normal condition (22 °C/20 °C, day/ night) or low temperature (15 °C/8 °C, day/ night) 12 h later. After 24 h of low temperature, these enzymes activities were measured. Data are expressed as the mean ± standard error of three independent biological replicates. The experiments were repeated for three times. Different letters above the bars indicate a significant difference determined by one-way ANOVA with Tukey’s test (*P <* 0.05). SOD, superoxide dismutase; CAT, catalase; APX, ascorbate peroxidase; MDHAR, monodehydroascorbate reductase; DHAR, dehydroascorbate reductase; GR, glutathione reductase

**Fig. 8:**
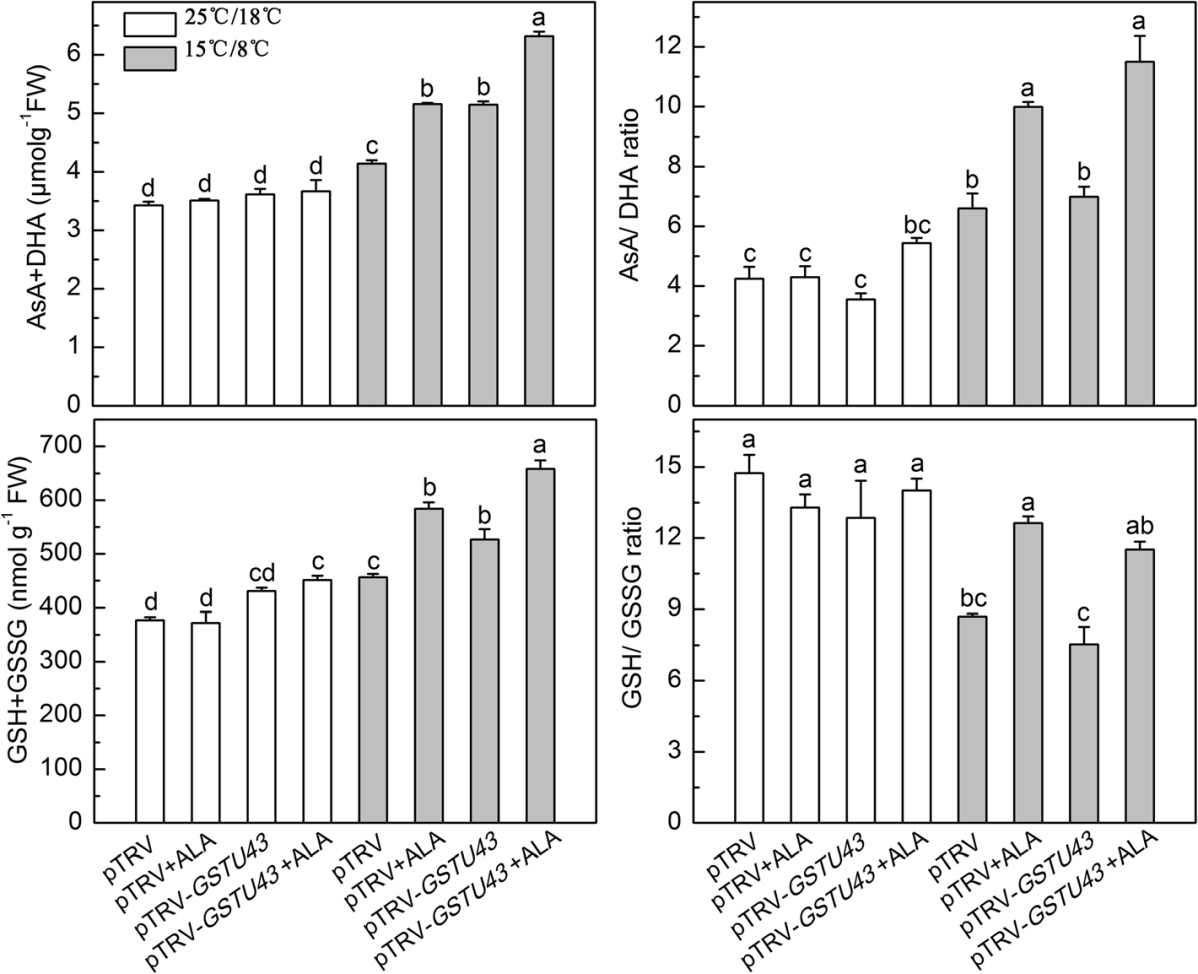
Effects of exogenous ALA on the glutathione and ascorbate content in partial *GSTU43*-silenced plants under low temperature. The VIGS plants leaves were treated with distilled water or 25 mg·L^− 1^ ALA, then exposed to normal condition (22 °C/20 °C, day/ night) or low temperature (15 °C/8 °C, day/ night) 12 h later. After 24 h of low temperature, the glutathione and ascorbate were measured. Data are expressed as the mean ± standard error of three independent biological replicates. The experiments were repeated for three times. Different letters above the bars indicate a significant difference determined by one-way ANOVA with Tukey’s test (*P <* 0.05). GSH, glutathione; GSSG, glutathione disulfide; AsA, ascorbate; DHA, dehydroascorbate

Under normal condition, pTRV-*GSTU43* plants compared to pTRV plants had much less CAT and GR activity, and ALA application slightly restored these activities (Fig. 7). Under low temperature, pTRV-*GSTU43* plants compared to pTRV plants had greater MDHAR and DHAR activity, and more ascorbate [measured as AsA and dehydroascorbate (DHA), combined], more glutathione [measured as GSH and glutathion disulfide (GSSG) combined], and slightly lower SOD, CAT, APX and GR activities, and slightly lower ratio of GSH/ GSSG (Figs. 7 and 8). When the low temperature stressed pTRV-*GSTU43* plants were supplied exogenous ALA, there was a significant increase in all these antioxidase activities and antioxidant contents, compared to the same plants without ALA treatment.

## Discussion

Plants live in a changeable environment, which requires them to cope with a variety of environmental stress [58]**.** Tomatoes are a warm weather crop, and during production in some climates, they may be exposed to low temperature in winter and early spring. This can negatively affect their growth and development [59]. Alleviating the plant cold tolerance in different periods via plant growth regulators is a feasible approach. For example, GABA improved cold tolerance of tea plants by inducing amino biosynthesis, and carbon and nitrogen metabolism [60]. Interestingly, recent research showed that melatonin application at grain filling in maternal plants could improve the wheat progeny seedling chilling tolerance by regulating the redox homeostasis and photosynthesis [61]. This study of the low temperature stress response of tomatoes was prompted in part by preliminary results that exogenous ALA improved the tolerance of tomatoes to low temperature by regulating GSH and AsA pools [12]. In addition, many studies have demonstrated that exogenous ALA enhances chlorophyll content of crops exposed to low temperature [6, 10, 62].

Previous studies showed that exogenous ALA promoted the content of chlorophyll precursors in *Arabidopsis* [63] and Kentucky bluegrass [64]. We observed this promoting effect of ALA in tomato, also, as shown in Fig. 2 for plants in normal temperature.

It has also been noted that when plants were exposed to stress, firstly, the membranes of stressed cells may be damaged by peroxides. And then, the free porphyrinogens leaked into the cytosol from the damaged membranes and were oxidized to the lipophilic and phytotoxic protoporphyrin, which may cause more serious oxidative damage [25, 29]. In addition to the damage of binding protein and key enzymes in chlorophyll synthesis which affected the chlorophyll synthesis [65], this may serve as a mechanism for the plants to protect themselves against the damaging effect of protoporphyrin. We suggest that this self-preservation mechanism may account for our observation that UROIII was not converted to Proto IX in low temperature-stressed plants, leading not only to reduce Proto IX, but to lower levels of ALA, Mg-Proto-IX, Pchl, and chlorophyll than in the plants at normal temperature (Figs. 1 and 2).

Application of ALA alleviated the effects of low temperature on chlorophyll synthesis, accompanied by elevation of the porphyrin level. It may be that the increased amount of porphyrin in the ALA-treated plants regulated nuclear gene expression, operating in signaling [66], which then enhances plant antioxidant capacity [67], and reduces membrane lipid peroxidation of the tomato under low temperature [12]. This would permit coordinated chlorophyll synthesis (Figs. 1 and 2). Based on our results with chlorophyll synthesis related genes expression, it may be that the ALA-mediated effects on chlorophyll biosynthesis are regulated at the translation or post-translational levels, but not at the transcriptional levels (Fig. 3) [55], which still needed further researches. Similar results were also reported for ALA-regulated chlorophyll synthesis in oilseed rape [8] and *Arabidopsis* [54]. Redox homeostasis is important for coordinated chlorophyll synthesis [54]. Studies have shown that GSTs regulate plant redox balance by improving its antioxidant and avoiding autoxidation during stress via conjugation of leaked porphyrinogens in the cytosol [19, 25, 68].

In this study, ALA treatment up-regulated the *GSTU43* gene and protein expression and GST activity (Fig. 4, Additional file 2: Figure S1). Inhibition of endogenesis ALA via GAB did not weaken the *GSTU43* gene and protein expression, and GST activity, on the contrary, illustrated a maximum value under both temperature. However, GAB plus ALA treatment declined these trends. This said, exogenous ALA triggered GST activity may be mainly related to the hormones signals or transcription factor, such as ERF and MYB [19], induced by exogenous ALA, but not completely dependent on endogenesis ALA. In other words, the mechanism by which the exogenous ALA activated *GSTU43* transcription and GST activity was different from that of the endogenous ALA. However, GAB, which may be as a harmful chemical inducer [19], had other effects than reducing the endogenous ALA level, and these other effects override the impact of the reduced ALA level on the expression of *GSTU43* and GST activity. Simultaneously, GAB stimulated high *GSTU43* transcript, and then provoked the GSTU43 protein expression and GST activity to cope with its own damage effects. Still, this was an interesting and confusing result, which needs further research.

Although low temperature alone induced GST activity, simultaneously decreased the activities of CAT and GR, and the ratio of reduced GSH, which eventually leads to the imbalance of redox and membrane lipid peroxidation (Figs. 5, 6, 7, and 8). The damaged cell membrane may influence the proteases which bind to the thylakoid membrane and catalyze the porphyrin synthesis. And porphyrins may partly leak from the chloroplasts [25], leading to a high free porphyrin level which triggered autooxidation and then further caused serious membrane damage at the same time (Fig. 5), and reduced plant growth (Additional file 3: Figure S2b and c). Exogenous ALA significantly increased the activity of GST, which may cooperate with AsA-GSH cycle to regulate the redox homeostasis, and also reduced membrane lipid peroxidation damage by binding the over-accumulation and extravasation of porphyrins [25]. This may provide the redox homeostasis for chlorophyll synthesis [54].

Silencing of *GSTU43* caused MDA accumulation, increased REC, and decreased the Fv/Fm. Exogenous ALA treatment reversed these effects in low temperature (Fig. 5). We considered that *GSTU43* participated in removal of ROS that accumulated during low temperature stress of these plants.

In plants, O_2_^−^ is rapidly converted into H_2_O_2_ via SOD [69], which is, in turn, converted to H_2_O or O_2_ by an AsA- and/or a GSH-regenerating cycle, and/or by CAT [70]. In present study, in low temperature, *GSTU43* expression and GST activity of pTRV-*GSTU34* plants compared to pTRV plants significantly decreased (Fig. 6), while the activities of SOD, CAT, APX, and GR were slightly decreased (Fig. 7). Apparently, low temperature stress caused ROS accumulation and membrane injury in pTRV-*GSTU43* plants, mainly due to the inhibition of GST activity, although reduced activities of SOD, CAT, APX, and GR may have also contributed.

The observed high levels of AsA + DHA in low temperature-stressed pTRV-*GSTU43* plants, compared with low temperature-stressed pTRV plants may have been due to the elevated activities of MDHAR and DHAR, which could reduce the DHA to AsA with the assistant of GSH, and ultimately caused the slight decline in the ratio of GSH/GSSG in pTRV-*GSTU43* plants (Figs. 7 and 8). pTRV-*GSTU43* plants plus exogenous ALA caused a dramatic enhancement of these enzyme activities, and increased the levels of GSH + GSSG and AsA + DHA, and the ratios of AsA/DHA and GSH/GSSG, compared to pTRV-*GSTU43* plants under low temperature (Figs. 6, 7, and 8). We suggest that ALA alleviated the oxidative stress in pTRV-*GSTU43* plants primarily through GST activity, with the cooperation of the AsA-GSH cycle, effecting redox balance for coordinated chlorophyll synthesis [54].

## Conclusions

Low temperature disturbed the redox balance, and damaged the integrity and stability of the membrane, which may eventually affected the chlorophyll synthesis, and reduced the chlorophyll content. Exogenous ALA increased GST protein expression and enzyme activity, encoded by *GSTU43*, and appeared to play a central role in protecting the tomato plant from low temperature-induced oxidative stress. It may operate with the assistance of the AsA- and/or a GSH-regenerating cycle, and actively regulated the plant redox homeostasis. This latter effect may, reduce the degree of membrane lipid peroxidation, which was essential for the coordinated synthesis of chlorophyll. Future studies may reveal the pathway how ALA induced GST outburst, which is crucial for increasing the plant’s tolerance of oxidation stress and maintain a stable chlorophyll synthesis under low temperature.

## Additional files

Additional file 1: **Table S1.** Gene-specific primers designed for qRT-PCR. (DOCX 16 kb)

Additional file 2: **Figure S1.** ALA induced changes of glutathione S-transferase (*GSTU43*) gene using RNA-seq in tomato leaves under low temperature. The tomato leaves treated with distilled water or 25 mg·L^− 1^ ALA then exposed to normal condition (control and ALA) or low temperature (LT and LTA) 12 h later. After 24 h low temperatures, the FPKM of *GSTU43 gene* we measured. Data are expressed as the mean ± standard error of three independent biological replicates. The experiments were repeated for three times. Different letters above the bars indicate a significant difference determined by one-way ANOVA with Tukey’s test (*P <* 0.05). (JPG 886 kb)

Additional file 3: **Figure S2.** Characterization of tomato pTRV-*GSTU43* plants and ALA induced changes of phenotypes and growth in *GSTU43*-silenced plants under low temperature. (a) Expression of *GSTU43* gene expression in pTRV and pTRV-*GSTU43* plants. (b) The dry weight of plants. (c) The phenotypes of plants. The *GSTU43* gene expression was measured at 35 d after infection. The gene transcription level in pTRV plants was normalized as 1. And then VIGS plants leaves were treated with distilled water or 25 mg·L^− 1^ ALA, subsequently exposed to normal condition (22 °C/20 °C, day/ night) or low temperature (15 °C/8 °C, day/ night) 12 h later. After 6 d of low temperature, the plants dry weight were measured and taken photos. Data are expressed as the mean ± standard error of seven independent biological replicates. The experiments were repeated for three times. Different letters above the bars indicate a significant difference determined by one-way ANOVA with Tukey’s test (*P <* 0.05), while ‘**’ above the bars indicate a significant difference determined by one-way ANOVA with Tukey’s test (*P <* 0.01). (JPG 3827 kb)

## Data Availability

The datasets analyzed during the current study are available from the corresponding author on reasonable request.

## Abbreviations

ALA: 5- aminolevulinic acid
APX: Ascorbate peroxidase
AsA: Ascorbate
CAT: Catalase
DHAR: Dehydroascorbate reductase
Fv/Fm: Maximal quantum yield of PSII photochemistry
GAB: Gabaculine
GR: Glutathione reductase
GSH: Glutathione
GST: Glutathione S-transferase
H_2_O_2_: Hydrogen peroxide
MDA: Malondialdehyde
MDHAR: Monodehydroascorbate reductase
Mg-proto IX: Mg-protoporphyrin IX
PBG: Porphobilinogen
Pchl: Protochlorophyll
Proto IX: Protoporphyrin IX
REC: Relative electrical conductivity
SOD: Superoxide dismutase
UROIII: uroporphyrinogen III
