# Supplementary material for: GSTU43 gene involved in ALA-regulated redox homeostasis, to maintain coordinated chlorophyll synthesis of tomato at low temperature
Source: BMC Plant Biol. 2019 Jul 18;19:323. doi: 10.1186/s12870-019-1929-1 (PMC6639942; doi:10.1186/s12870-019-1929-1)
Supplement: Supplementary file 1 — Table S1. Gene-specific primers designed for qRT-PCR. (DOCX 16 kb) [file 12870_2019_1929_MOESM1_ESM.docx]

Table S1. Gene-specific primers designed for qRT-PCR.

| **Gene name** | **Accession numbers** | **Forward primer** | **Reverse primer** |
| --- | --- | --- | --- |
| *actin7* | NM001308447 | GGGATGGAGAAGTTTGGTGGTGG | CTTCGACCAAGGGATGGTGTAGC |
| *GAPDH* | NM001247874 | ACCACAAATTGCCTTGCTCCCTTG | ATCAACGGTCTTCTGAGTGGCTGT |
| *GSTU43* | NM001247157 | AGAGGAGTCAAACAAAGCTAAAGAG | ATTCCCATCCAATATGCCATC |
| *ALAD* | XM010326888 | TTGAGAAAGATGGGGTTGACTG | GCTGCTGACGACCTACGATTAC |
| *PBGD* | XM004243933 | TTCTTCGCCTTGCCGTAAAT | CTTCCTCGTGTTCCAACTCTGAT |
| *UROD* | XM004240802 | CTTGATGCTGTTCGGGGTAA | CAAAAGGGATGTTCATACCAGAG |
| *CPOX* | XM0042483644 | GGAAACCCCAGAAACTGAACG | ACCCTACTAATCCCACCACCC |
| *PPOX* | NM001348379 | GCAATGGCTGGAGAACACG | TTCCTGCTCCGACTACCACA |
| *CHLM* | XM004235797 | ACCTTAAACCCTAATCCCCAAC | TGCCGCCACTGAACTACCT |
| *POR* | XM004251804 | ACGGTGGTGACTTTGATGGTG | AAAGGAGCCTAAACAAGGGGAT |
| *CHLG* | XM004246270 | TTTCAACTACAACAACCCACTTCG | CGTTTCTTGCTTGGCTCCTTT |
| *CAO* | XM004250263 | GAGGGTGGGATTCCAGATAAGA | AACCTTTTCGTGTAGCAGCATT |
