# Supplementary figures and images for: GSTU43 gene involved in ALA-regulated redox homeostasis, to maintain coordinated chlorophyll synthesis of tomato at low temperature
Source: BMC Plant Biol. 2019 Jul 18;19:323. doi: 10.1186/s12870-019-1929-1 (PMC6639942; doi:10.1186/s12870-019-1929-1)

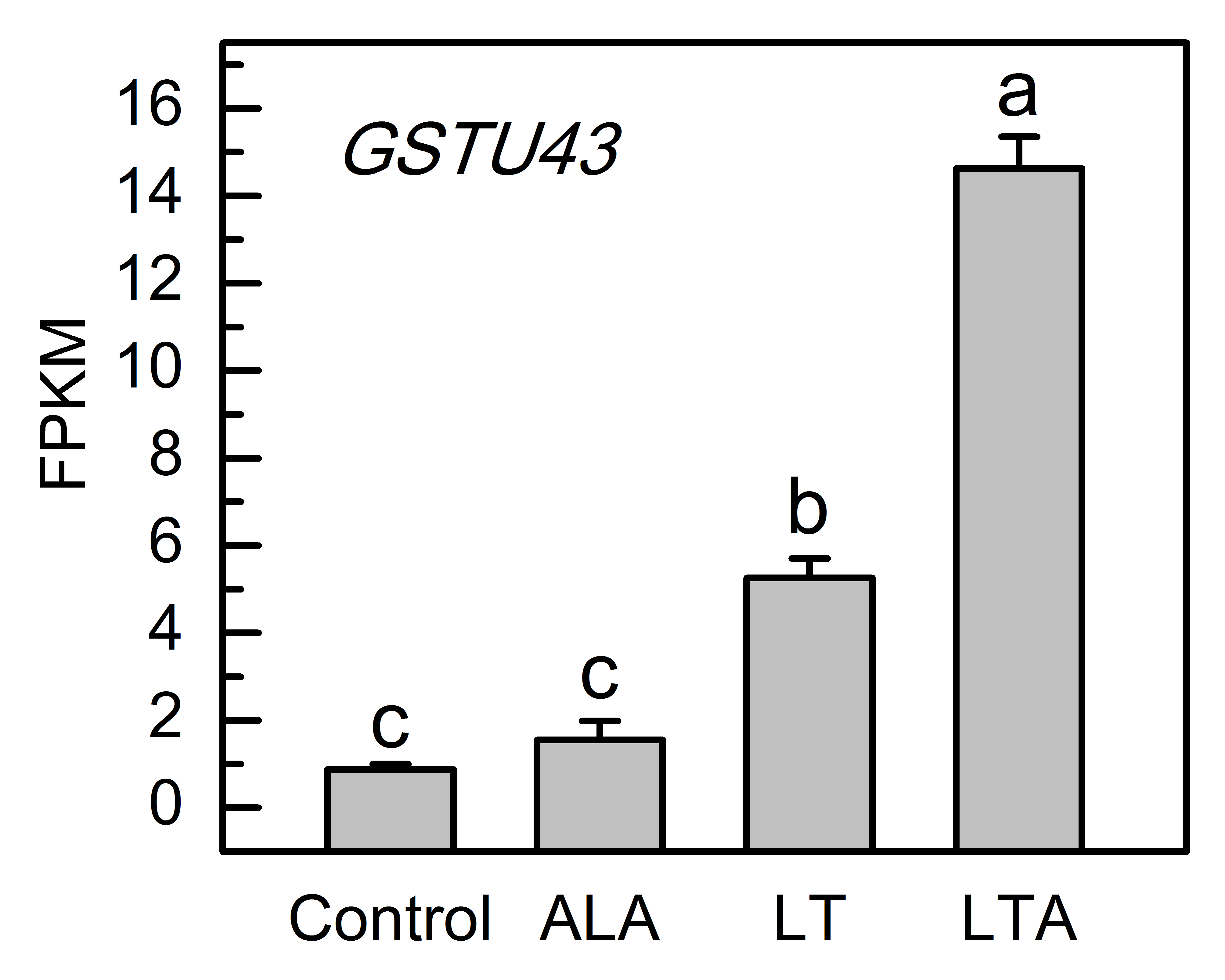

Supplement: Supplementary file 2 — Figure S1. ALA induced changes of glutathione S-transferase (GSTU43) gene using RNA-seq in tomato leaves under low temperature. The tomato leaves treated with distilled water or 25 mg·L− 1 ALA then exposed to normal condition (control and ALA) or low temperature (LT and LTA) 12 h later. After 24 h low temperatures, the FPKM of GSTU43 gene we measured. Data are expressed as the mean ± standard error of three independent biological replicates. The experiments were repeated for three times. Different letters above the bars indicate a significant difference determined by one-way ANOVA with Tukey’s test (P < 0.05). (JPG 886 kb) [file 12870_2019_1929_MOESM2_ESM.jpg]

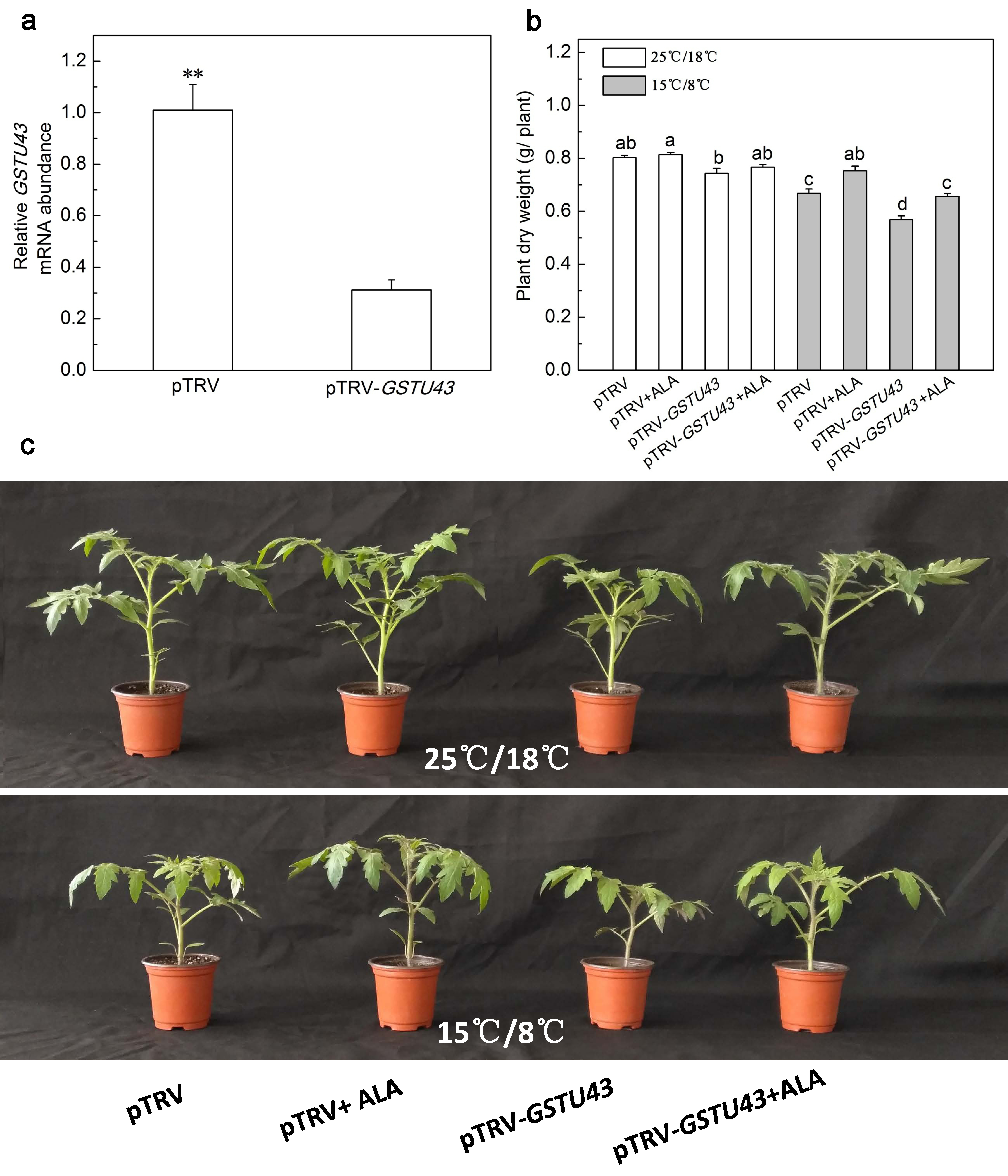

Supplement: Supplementary file 3 — Figure S2. Characterization of tomato pTRV-GSTU43 plants and ALA induced changes of phenotypes and growth in GSTU43-silenced plants under low temperature. (a) Expression of GSTU43 gene expression in pTRV and pTRV-GSTU43 plants. (b) The dry weight of plants. (c) The phenotypes of plants. The GSTU43 gene expression was measured at 35 d after infection. The gene transcription level in pTRV plants was normalized as 1. And then VIGS plants leaves were treated with distilled water or 25 mg·L− 1 ALA, subsequently exposed to normal condition (22 °C/20 °C, day/ night) or low temperature (15 °C/8 °C, day/ night) 12 h later. After 6 d of low temperature, the plants dry weight were measured and taken photos. Data are expressed as the mean ± standard error of seven independent biological replicates. The experiments were repeated for three times. Different letters above the bars indicate a significant difference determined by one-way ANOVA with Tukey’s test (P < 0.05), while ‘**’ above the bars indicate a significant difference determined by one-way ANOVA with Tukey’s test (P < 0.01). (JPG 3827 kb) [file 12870_2019_1929_MOESM3_ESM.jpg]
